# An alternative to the Cauchy distribution

**DOI:** 10.1016/j.mex.2019.02.025

**Published:** 2019-03-22

**Authors:** Ayman Alzaatreh

**Affiliations:** Department of Mathematics and Statistics, American University of Sharjah, Sharjah, United Arab Emirates

**Keywords:** The paper proposes an alternative to the Cauchy distribution using the T-X family framework proposed by Alzaatreh et al. (2013). The proposed distribution can be left skewed, right skewed or symmetric. The moments are defined under some restriction on the parameter space, Estimation, Moments, T-X family, Exponentiated-exponential-X family, Shannon entropy, Cauchy distribution

## Abstract

A few generalizations of the Cauchy distribution appear in the literature. In this paper, a new generalization of the Cauchy distribution is proposed, namely, the exponentiated-exponential Cauchy distribution (EECD). Unlike the Cauchy distribution, EECD can have moments for some restricted parameters space. The distribution has wide range of skewness and kurtosis values and has a closed form cumulative distribution function. It can be left skewed, right skewed and symmetric. Two different estimation methods for the EECD parameters are studied.

• A new generalization of the Cauchy distribution is proposed, namely, exponentiated-exponential Cauchy distribution (EECD).
• EECD has flexible shape characteristics. Moreover, EECD moments are defined under some restrictions on the parameter space.

**Specifications Table**

**Table:** 

**Subject Area:**	*Mathematics*
**More specific subject area:**	*Statistics, Distribution Theory*
**Method name:**	*The paper proposes an alternative to the Cauchy distribution using the T-X family framework proposed by Alzaatreh et al. (2013). The proposed distribution can be left skewed, right skewed or symmetric. The moments are defined under some restriction on the parameter space.*
**Name and reference of original method:**	*Alzaatreh, A., Lee, C. & Famoye, F. (2013). A new method for generating* *families of continuous distributions. Metron, 71, 63-79.*
**Resource availability:**	*Data source is mentioned in the paper*

## Method details

The Cauchy distribution was first appeared in works of Pierre de Fermat and then studied by many researchers such as Isaac Newton, Gottfried Leibniz and others (see Ref. [1]). The Cauchy density was also used by Poisson [2] as counterexamples for some general results in probability. Based on Johnson et al. [1], the Cauchy distribution becomes associated with Cauchy [3] when Cauchy responded to an article by Bienayme’ [4] criticizing a method of interpolation proposed by Cauchy.

The fact that the Cauchy distribution has no moments, and therefore the law of large numbers does not apply, motivates researchers to generalize the Cauchy distribution. Few generalizations of the Cauchy distribution have appeared in the literature; Rider [5] proposed and study a generalization of the Cauchy distribution, Batschelet [6] proposed the wrapped-up Cauchy distribution, the skew-Cauchy distribution was proposed by Arnold and Beaver [7], another class of skew-Cauchy distribution was studied by Behboodian et al. [8], Huang and Chen [9] proposed a generalization of the skew-Cauchy distribution and recently Alshawarbeh et al. [10] used the beta family introduced by Eugene et al. [11] to generate the so called beta-Cauchy distribution.

In this paper, a new generalization of the Cauchy distribution is proposed. The proposed distribution is very flexible in terms of shapes, it can be left skewed, right skewed or symmetric. The moments are defined for some restricted values of the parameters. Also, the proposed distribution has a closed form cumulative distribution function (CDF) which adds more advantage to this distribution. The simplicity of the proposed distribution (closed from CDF) and the great flexibility in modeling real life data (see Application) will attract researchers to use this distribution as an alternative of the Cauchy distribution in modeling different scenarios.

Let r(t) be the probability density function (PDF) of a random variable T∈[a,b], for −∞≤a<b≤∞. Let W(.):[0,1]→ℜ be a link function satisfies the following conditions:

(1.1) $$\left(\begin{array}{l} W(.)\;\text{is}\;\text{absolutely}\;\text{continuous}\;\text{and}\;\text{monotonically}\;\text{non-decreasing}\;\text{function}\\ W(0)\to a\;\text{and}\;W(1)\to b. \end{array}\right\}$$

The CDF of the *T*-*X* family of distributions defined by Alzaatreh et al. [12] is given by

(1.2) $$G(x)=\int_{a}^{W\left(F(x)\right)}r(t)dt,$$

where W(.) satisfies the conditions in (1.1).

The corresponding PDF associated with (1.2) is

(1.3) $$g(x)=\left\{\frac{d}{dx}W\left(F(x)\right)\right\}r\left\{W\left(F(x)\right)\right\}.$$

If $W(F(x))=-\log\left(1-F(x)\right)$, then W(.) satisfies the conditions (1.1) and (1.3) reduces to

(1.4) $$g(x)=\frac{f(x)}{1-F(x)}r\left(-\log\left(1-F(x)\right)\right)=h(x)\;r\left(H(x)\right),$$

where h(x)=f(x)/(1−F(x)) and H(x)=−log(1−F(x)) are, respectively, the hazard and cumulative hazard functions associated with f(x). Some general properties of the *T*-*X* in (1.4) have been recently studied, for more details see Alzaatreh et al. [12,13,31] and Lee et al. [14]. Also the discrete analogue of the *T*-*X* family is studied by Alzaatreh et al. [15].

If a random variable *T* follows the exponentiated exponential distribution (EED) with parameters α and λ, $r(t)=\lambda \alpha \;e^{-\lambda t}{\left(1-e^{-\lambda t}\right)}^{\alpha -1},\;\;\;t\geq 0$, the definition in (1.4) leads to the exponentiated exponential-*X* family (*EE*-*X*) with the PDF

(1.5) $$g_{F}(x)=\alpha \lambda f(x){\left(1-{\left(1-F(x)\right)}^{\lambda }\right)}^{\alpha -1}{\left(1-F(x)\right)}^{\lambda -1}.$$

When α=1 and λ=n where *n* is a positive integer, the *EE*-*X* family in (1.5) reduces to the distribution of the first order statistics, $X_{\left(1\right)}$, from a sample of size *n* from f(x). When α=n and λ=1, the *EE*-*X* family reduces to the distribution of the *n*^th^ order statistics, $X_{\left(n\right)}$, from a sample of size *n* from f(x). When α=1, the *EE*-*X* reduces to the exponentiated 1−F(x) distribution with parameter λ. The parameters α and λ controls the skewness and kurtosis of the family. Also, as x→−∞, $g_{F}∼\alpha \lambda^{\alpha }f\;F^{\alpha -1}$ and as x→∞, $g_{F}∼\alpha \lambda f\;{\left(1-F\right)}^{\lambda -1}$.

The paper is outlined as follows. First we define using (1.5) a new generalization of the Cauchy distribution, namely, the exponentiated-exponential Cauchy (EEC) distribution. Then we study some properties of EEC distribution including quantile skeweness and kurtosis, Shannon entropy and moments. Also, different characterizations of the EE-X family based on truncated moments are discussed. Parameter Estimation deals with estimation methods of the EEC distribution. Applications of the EEC distribution to real data sets are provided.

## The exponentiated-exponential Cauchy distribution

If *X* is a Cauchy random variable with parameter θ>0, then $f(x)={\left(\pi \theta (1+{\left(x/\theta \right)}^{2})\right)}^{-1},\;$ −∞<x<∞, and $F(x)=\frac{1}{2}+\frac{1}{\pi }\tan^{-1}\left(x/\theta \right)$, then (1.5) reduces to

(2.1) $$g(x)=\frac{\alpha \lambda }{\pi \theta [1+{\left(x/\theta \right)}^{2}]}{\left(1-{\left(0.5-\pi^{-1}\tan^{-1}(x/\theta )\right)}^{\lambda }\right)}^{\alpha -1}{\left(0.5-\pi^{-1}\tan^{-1}(x/\theta )\right)}^{\lambda -1},x\in ℜ.$$

A random variable *X* with the PDF *g*(*x*) in (2.1) is said to follow the exponentiated-exponential Cauchy distribution and will be denoted by EEC $\left(\alpha ,\lambda ,\theta \right)$. When λ=1, the EEC distribution reduces to the exponentiated Cauchy distribution defined by Sarabia and Castillo [16]. When α=λ=1, the EEC distribution reduces to the Cauchy distribution with parameters θ. Therefore, the density in (2.1) is a generalization of the Cauchy density. From (2.1), we obtain the CDF of the EEC distribution as

(2.2) $$G(x)={\left(1-{\left(0.5-\pi^{-1}\tan^{-1}(x/\theta )\right)}^{\lambda }\right)}^{\alpha },\;x\in ℜ.$$

The hazard function associated with the EEC distribution is

(2.3) $$h(x)=\frac{g(x)}{1-G(x)}=\frac{\alpha \lambda {\left(1-{\left(0.5-\pi^{-1}\tan^{-1}(x/\theta )\right)}^{\lambda }\right)}^{\alpha -1}{\left(0.5-\pi^{-1}\tan^{-1}(x/\theta )\right)}^{\lambda -1}}{\pi \theta \left(1+{\left(x/\theta \right)}^{2}\right)\left[1-{\left(1-{\left(0.5-\pi^{-1}\tan^{-1}(x/\theta )\right)}^{\lambda }\right)}^{\alpha }\right]},\;x\in ℜ.$$

A physical interpretation of the EECD in (2.2) is possible for integer values of α and λ. For example, let us start with a system ℱ which consists of α independent components say $X_{i},\;\;i=1,\cdots ,\alpha$. Suppose that each component consists of λ subcomponents. Assume that the system ℱ fails if all of the α components fail (i.e. parallel system with respect to α components). Assume further that each of the α components fails if at least one of the λ subcomponents fails. Now, let *X* denote the lifetime of the system ℱ and $X_{i1},\cdots ,X_{i\lambda },\;i=1,\cdots ,\alpha$ be independent random variables follow Cauchy (θ). Then the CDF of *X* can be computed as follows

$$\begin{array}{cc} G_{X}(x)=P(X_{1}\leq x,\cdots ,X_{\alpha }\leq x) & \begin{array}{c} ={\left(1-P(X_{1}>x)\right)}^{\alpha }\\ ={\left(1-{\left(1-P(X_{11}\leq x)\right)}^{\lambda }\right)}^{\alpha }\\ ={\left(1-{\left(1-P(X_{11}\leq x)\right)}^{\lambda }\right)}^{\alpha }={\left(1-{\left(0.5-\pi^{-1}\tan^{-1}(x/\theta )\right)}^{\lambda }\right)}^{\alpha }. \end{array} \end{array}$$

In the next section, some general properties of the EEC distribution will be addressed including transformations, limiting behavior, quantile function and the Shannon entropy.

Remark 1 The connection of EEC with some known distributions can be seen as follows; If a random variable *Y* follows Kumaraswamy’s distribution with parameters α and λ, then the random variable X=θcot(πY) follows EEC $\left(\alpha ,\lambda ,\theta \right)$. Also, if a random variable *Y* follows beta distribution with shape parameters a=1 and b=α, then the random variable $X=\theta \cot(\pi Y^{1/\lambda })$ follows EEC $\left(\alpha ,\lambda ,\theta \right)$. Finally, if a random variable *Y* follows exponentiated-exponential distribution with parameters α and λ, then the random variable $X=\theta \cot(\pi e^{-Y})$ follows EEC $\left(\alpha ,\lambda ,\theta \right)$.

The mode of the EEC distribution is the solution of k(w)=0 where

(2.4) $$k(w)=(1-w^{\lambda })(1-\lambda \alpha -2\pi w\cot(\pi w))+\lambda (\alpha -1),\;\;w\in (0,1)$$

and $w=\frac{1}{2}-\frac{1}{\pi }\tan^{-1}(x/\theta )$. To find the mode of the EECD; first find $w_{0}$ such that $k(w_{0})=0$ and then obtain the mode at $x_{0}=\theta \cot(\pi w_{0})$. If α=λ=1, then (2.4) implies that $x_{0}=0$ is the only mode. This result agrees with the mode of the standard Cauchy distribution.

Theorem 1 If λα≥1 then EECD is unimodal.

Proof To show EECD is unimodal, it suffices to show that k(w)=0 in (2.4) has a unique solution on the interval (0, 1). First notice that $\lim_{w↑0}k(w)=-1-\lambda <0$ and $\lim_{w↓1}k(w)=\lambda (\alpha +1)>0$. Since k(w) is continuous on (0, 1), k(w)=0 has at least one solution. Now let ψ(w)=a(w)b(w) where $a(w)=1-w^{\lambda }$ and b(w)=−2πwcot(πw). Claim: ψ(w)↗ on (0, 1). It is clear that a(w)↘ and a(w)>0 on (0, 1). Also, $b^{\prime }(w)=2\pi (\pi w\csc^{2}(\pi w)-\cot\pi w)$. The fact that sinπw<πw,  w∈(0,1) implies that $\pi w\csc^{2}(\pi w)>\frac{1}{\pi w}$. Therefore, b(w)>2π c(w) where $c(w)=\frac{1}{\pi w}-\cot\pi w$. Furthermore, $c^{\prime }(w)=\frac{-1}{\pi w^{2}}+\pi \csc^{2}(\pi w)>\frac{-1}{\pi w^{2}}+\frac{1}{\pi w^{2}}=0$. Since $\lim_{w↑0}c(w)=0$ and $\lim_{w↓1}c(w)=\infty$, we get c(w)>0. This implies that b(w)↗ on (0,1). Now in order to show ψ(w) w∈(0,1) on (0,1), note that if 0<w≤1.5 we have b(w)<0. And since $a^{\prime }(w)<0$, $b^{\prime }(w)>0$ and a(w)>0 for all w∈(0,1), we get $\psi^{\prime }(w)>0$. For the case 0.5<w<1, it is not difficult to show $\psi^{\prime }(w)>0$ and hence ψ(w)↗ on (0, 1). This ends the proof of the claim. Now,

k(w)=a(w)(1−λα+b(w))+λ(α−1)=(1−λα)a(w)+ψ(w)+λ(α−1). If λα≥1 and a(w)↘ then (1−λα)a(w) ↗. This implies that (1−λα)a(w)+ψ(w) is strictly increasing and hence k(w) is strictly increasing function. Since k(w)=0 has at least one solution, k(w)=0 must have a unique solution on (0, 1) □

**Fig. 1:**
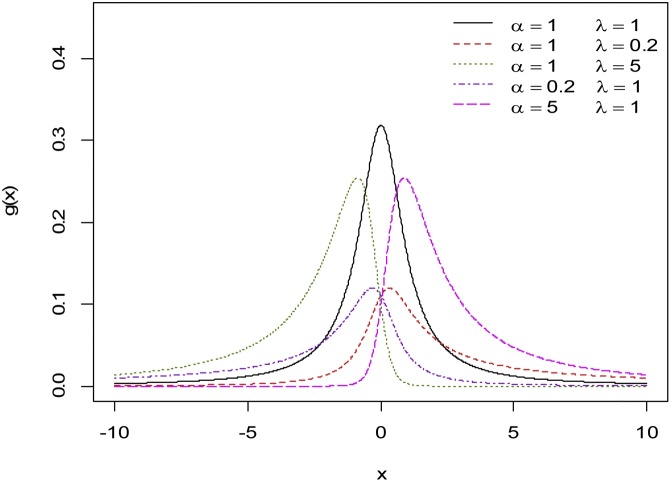
The EEC PDF for various values of *α* and λ.

**Fig. 2:**
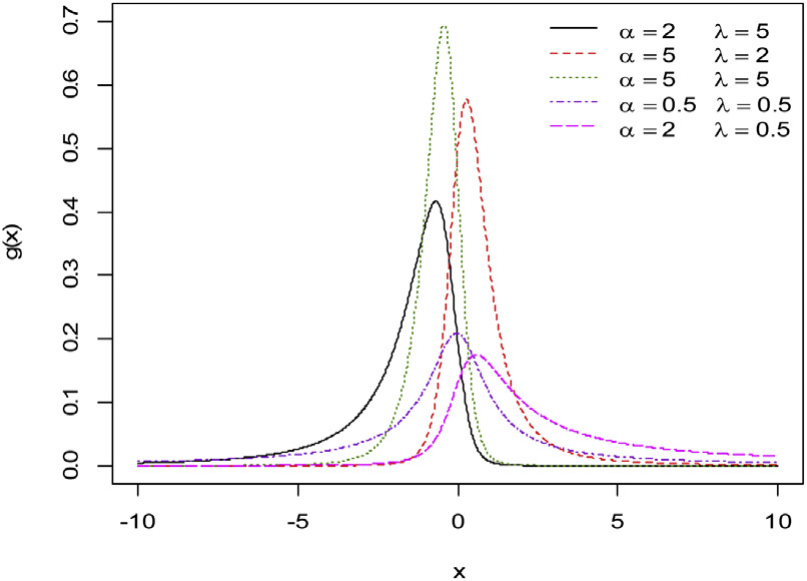
The EEC PDF for various values of *α* and λ.

It is not straightforward to show analytically that the equation k(w)=0 has a unique solution for the case λα<1. However, from Fig. 1, Fig. 2 it appears that the EECD is a unimodal distribution.

**Fig. 3:**
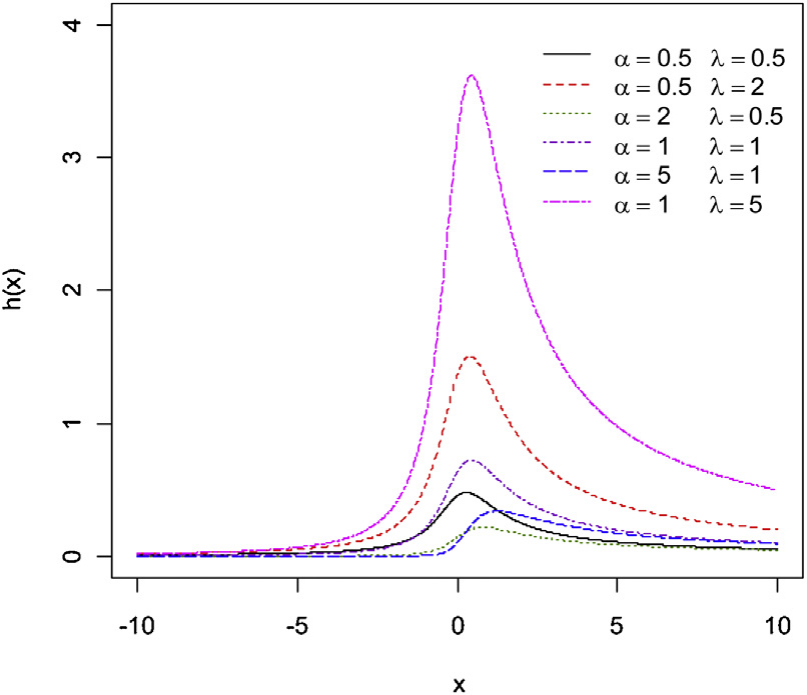
The EEC hazard function for various values of *α* and λ.

In Fig. 1, Fig. 2, Fig. 3, various graphs of g(x) and h(x) are provided where the scale parameter θ=1. These plots indicate that the EEC PDF possesses great flexibility in terms of shapes; it can be symmetric, right skewed and left skewed. For fixed values of λ, the skewness (towards the left) of the distribution increases as α increases. Also, for fixed values of α, the skewness (towards the right) of the distribution increases as λ increases. Furthermore, Fig. 2 shows that the distribution is left skewed (right skewed) whenever α≥1 (α<1) and λ<1 (λ≥1). The plots in Fig. 3 indicate that the EEC hazard function shape is always upside-down bathtub.

Remark 2 Let Q(p),  0<p<1 denote the quantile function for the EECD. Then, Q(p) is given by

(2.5) Q(p)=θcotπ1−p1α1λ.

To explore the effect of the shape parameters when the quantile function is in closed form, Alzaatreh et al. [12] suggested using the quantile based Skewness and kurtosis for the *T*-*X* family of distributions. The measure of skewness *S* defined by Galton [17] and the measure of kurtosis *K* defined by Moors [18] are given by

(2.6) $$S=\frac{Q(6/8)-2Q(4/8)+Q(2/8)}{Q(6/8)-Q(2/8)}\;\text{and}\;K=\frac{Q(7/8)-Q(5/8)+Q(3/8)-Q(1/8)}{Q(6/8)-Q(2/8)}.$$

**Fig. 4:**
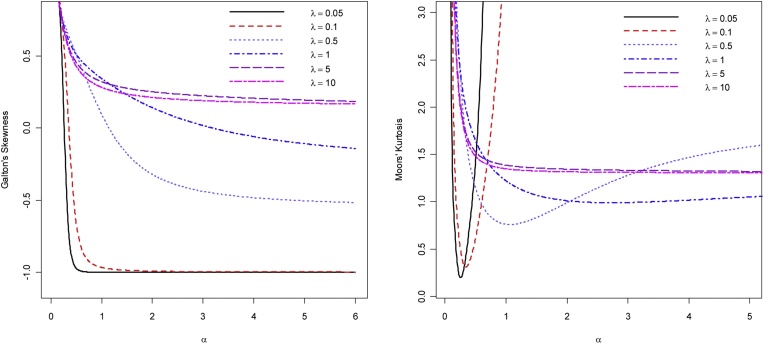
Galton’s Skewness and Moors’ Kurtosis for the EECD for various values of *α* and λ.

When the distribution is symmetric, *S* = 0 and when the distribution is right (or left) skewed, *S* > 0 (or < 0). As *K* increases, the tail of the distribution becomes heavier. Since the CDF of the ECC distribution is in closed form, equations in (2.6) are used to obtain the Galtons' skewness and Moors' kurtosis where the quantile function is defined in (2.5). Fig. 4 displays the Galton's skewness and Moors' kurtosis for the EECD when θ=1. From Fig. 4, the EECD takes wide range of skewness and kurtosis values. This indicates that the EECD can be very effective in modeling real data sets with various skewness and kurtosis values.

**Fig. 5:**
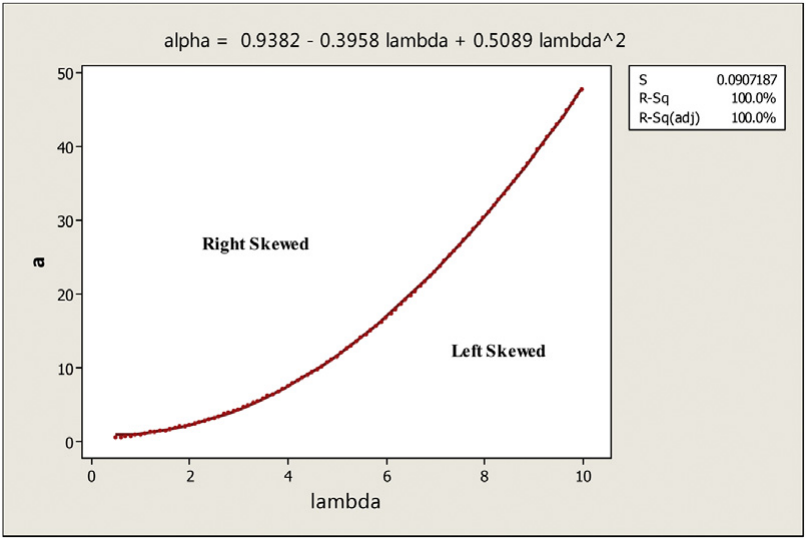
Skewness regions for the EECD when θ=1.

Galtons’ skewness is also used to determine the regions in which the EEC distribution is left skewed or right skewed. A numerical method is used to determine the points where the Galtons’ skewness equals to zero. Fig. 5 shows the regions in which the EEC distribution is left skewed or right skewed. The quadratic function in Fig. 5 connects the points where EEC distribution is symmetric.

## Some properties of EEC distribution

The entropy of a random variable *X* is a measure of variation of uncertainty [19].

Shannon entropy [20] for a random variable *X* with PDF *g*(*x*) is defined as $E\left\{-\log\left(g(X)\right)\right\}$. Since 1948, Shannon entropy has been used in many fields such as communication theory, engineering, physics and biology. Alzaatreh et al. [12] derived the Shannon entropy for the *T*-*X* family of distributions. Also, Ghosh and Alzaatreh [21] derived the Shannon entropy for the exponentiated exponential-*X* in (1.5) as

(3.1) $$\eta_{X}=-E\left\{\log f\left(F^{-1}\left(1-e^{-T}\right)\right)\right\}-\log(\lambda \alpha )+(1-1/\lambda )(\psi (\alpha )-\psi (1))-{\left(\alpha \lambda \right)}^{-1}+1,$$

where ψ is the digamma function and *T* is the exponentiated-exponential random variable with parameters α and λ. In the following theorem, we derive the Shannon entropy for the EEC distribution.

Theorem 2 The Shannon entropy for the EEC distribution is given by

(3.2) $$\eta_{X}=\log\left(\frac{\theta }{\alpha \lambda \pi }\right)+(1+1/\lambda )H(\alpha )-\alpha^{-1}+1-2\alpha \overset{\infty }{\underset{k=1}{\sum }}v_{k}B(\alpha ,2k\lambda^{-1}+1),$$

where H(α) is the harmonic number of α.

Proof We first need to find $-E\left\{\log f\left(F^{-1}\left(1-e^{-T}\right)\right)\right\}$, where f(x) and F(x) are, respectively, the PDF and the CDF of the Cauchy distribution. It is easy to show that $\log f(F^{-1}(1-e^{-T}))=-\log(\pi \theta )+2\log(\sin(\pi e^{-T}))$ and hence,

(3.3) $$-E\left\{\log f\left(F^{-1}\left(1-e^{-T}\right)\right)\right\}=\log(\pi \theta )-2\alpha \lambda \int_{0}^{\infty }\log(\sin(\pi e^{-t})){\left(1-e^{-\lambda t}\right)}^{\alpha -1}e^{-\lambda t}dt.$$

Now, using Gradshteyn and Ryzhik ([22], p. 55), log(sinπx) can be written as

(3.4) $$\log(\sin\pi x)=\log(\pi x)+\sum_{k=1}^{\infty }\underset{v_{k}}{\underset{︸}{\frac{{\left(-1\right)}^{k}{\left(2\pi \right)}^{2k}B_{2k}}{2k(2k)!}}}x^{2k},$$

where $B_{n}$ is the Bernoulli number.

By letting $u=e^{-t}$ and using the series representation of log(sinπx) in (3.4), (3.3) reduces to

(3.5) $$-E\left\{\log f\left(F^{-1}\left(1-e^{-T}\right)\right)\right\}=\log(\pi \theta )-2\alpha \lambda \int_{0}^{1}\log\left(\pi u\right)u^{\lambda }{\left(1-u^{\lambda }\right)}^{\alpha -1}du-2\alpha \sum_{k=1}^{\infty }v_{k}\;B(\alpha ,2k\lambda^{-1}+1),$$

where B(a,b)=Γ(a)Γ(b)/Γ(a+b), the beta function. Now,

$\int_{0}^{1}\log(u)u^{\lambda -1}{\left(1-u^{\lambda }\right)}^{\alpha -1}du=\frac{1}{\lambda^{2}}{\left(\frac{d}{dx}B\left(x,\alpha \right)\right|}_{x=1}=\frac{-1}{\lambda^{2}\alpha }H(\alpha )$. The results in (3.1) followed by using the above result and substituting (3.5) in (3.2) and using the fact that H(α)=ψ(α+1)−ψ(1). □

### Moments

On using Remark 1, the *r*th moments for the EEC distribution can be written as

(3.6) $$E(X^{r})=\alpha \lambda \theta^{r}\int_{0}^{1}\cot^{r}(\pi u){\left(1-u^{\lambda }\right)}^{\alpha -1}u^{\lambda -1}du.$$

By using (3.4) and the fact that $\cot(\pi u)=\frac{1}{\pi }\frac{d}{du}\log(\sin\pi u)$, one can obtain a series expansion for the cot(πu) as

(3.7) $$\cot(\pi u)=\sum_{k=0}^{\infty }\underset{w_{k}}{\underset{︸}{\frac{{\left(-1\right)}^{k}2^{2k}\pi^{2k-1}B_{2k}}{(2k)!}}}u^{2k-1}.$$

Therefore, ([22], p. 17),

(3.8) $$\cot^{r}(\pi u)=\sum_{k=0}^{\infty }v_{k}u^{2k-r},$$

where $v_{0}=\pi^{-r}$, $v_{m}=\pi m^{-1}\sum_{k=1}^{m}(kr-m+k)w_{k}v_{m-k},\;\;m\geq 1$

Hence, from (3.6) the *r*th moments for the EEC distribution can be written as

(3.9) $$E(X^{r})=\alpha \theta^{r}\sum_{k=0}^{\infty }v_{k}B[\alpha ,\lambda^{-1}(2k-r)+1].$$

The *r*th moments of the EEC do not always exist. The following theorem gives a necessary and sufficient condition for the existence of the *r*th moments of the EEC distribution.

Theorem 3 The *k*th moments of the EEC distribution exist if and only if both α and λ greater than *k*.

Proof Consider the following integrals

$$E(X^{k})=\int_{-\infty }^{-1}x^{k}g(x)dx+\int_{-1}^{1}x^{k}g(x)dx+\int_{1}^{\infty }x^{k}g(x)dx$$

where g(x) is defined in (2.1).

Without loss of generality assume θ=1. Since the middle integral above exists, it suffices to investigate the existence of the first and third integrands. Let $\delta (x)=\alpha \lambda \pi^{-\lambda }x^{-(\lambda -k+1)}$ for x≥1. Then $\int_{1}^{\infty }\delta (x)dx$ exists iff λ>k. One can easily show that $x^{k}g(x)∼\delta (x)$ as x→∞ and hence $\int_{1}^{\infty }x^{k}g(x)dx$ exists iff λ>k. Similarly one can show $\int_{-\infty }^{-1}x^{k}g(x)dx$ exists iff α>k. □

**Fig. 6:**
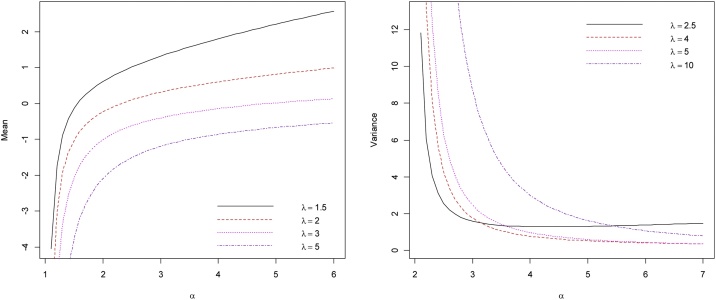
Mean and variance of the EECD for various values of *α* and λ.

Fig. 6 provides the mean and variance of the EEC distribution when the scale parameter θ=1 and for various combinations of α and λ. From Fig. 6 it appears that for fixed λ, the mean is an increasing functions of α while the variance is a decreasing function of α. Also, for fixed α, the mean is a decreasing function of λ.

The *r*th incomplete moments for the EEC distribution is defined as

(3.10) $$I_{r}(m)=\int_{-\infty }^{m}x^{r}g(x)dx.$$

On using the substitution $u=0.5-\pi^{-1}\tan^{-1}(x/\theta )$, (3.10) can be written as

(3.11) $$I_{r}(m)=\alpha \lambda \theta^{r}\int_{0}^{u_{m}}\cot^{r}(\pi u){\left(1-u^{\lambda }\right)}^{\alpha -1}u^{\lambda -1}du,$$

where $u_{m}=0.5-\pi^{-1}\tan^{-1}(m/\theta )$.

By using (3.8), (3.11) reduces to

(3.12) $$I_{r}(m)=\alpha \theta^{r}\sum_{k=0}^{\infty }v_{k}B_{1-u_{m}^{\lambda }}[\alpha ,\lambda^{-1}(2k-r)+1],$$

where $B_{x}(a,b)=\int_{0}^{x}u^{a-1}{\left(1-u\right)}^{b-1}du$, is the incomplete beta function.

The first incomplete moment is used to find the deviations from the mean and median. The deviation from the mean and the deviation from the median are used to measure the dispersion and the spread in a population from the center. The mean deviation from the mean is denoted by D(μ), and the mean deviation from the median *M* is denoted by D(M).

Corollary 1 The D(μ) and D(M) for EEC distribution are

$$D(\mu )=2\mu G(\mu )-2I_{1}(\mu )\;\text{and}\;D(M)=\mu -2I_{1}(M),$$

where $I_{1}(m)=\alpha \theta \sum_{k=0}^{\infty }v_{k}B_{1-u_{m}^{\lambda }}(\alpha ,\lambda^{-1}(2k-1)+1),\;\;\;\lambda >1$ and G(μ) can be found from Eq. (2.2).

Proof By definitions of D(μ) and D(M), it is easy to see that

$D(\mu )=E(X-\mu )=2\mu G(\mu )-2\int_{\;-\infty }^{\mu }xg(x)dx$ and $D(M)=E(X-M)=\mu -2\int_{\;-\infty }^{\;M}xg(x)dx$. The rest of the proof follows from (3.12). □

### Some characterizations of the *EE*-*X* family based on truncated moments

Glänzel [25] provides characterizations based on the truncated moments for some important distributions including the standard Cauchy distribution. For more information, one is referred to Hamedani [26]. Below, we provide some results from Glänzel [25] which will be used to show Theorem 5.

Theorem 4 Let X:Ω→H be a continuous random variable and let $g_{1}$ and $g_{2}$ be two functions defined on H=[a,b] for −∞≤a<b≤∞ such that $e_{g_{1}}(x)=e_{g_{2}}(x)\eta (x)$, where $e_{g_{i}}(x)=E(g_{i}(x)|X\geq x)$. Assume that $g_{1},g_{2}\in C^{1}(H)$, $g_{1}/g_{2}$ is of bounded variation and η(x) is continuously differentiable on *H*. Further assume that the equation $\eta g_{2}-g_{1}=0$ has no solution on the interior of *H*. Finally, assume that $\int_{a}^{b}\frac{\eta {g^{\prime }}_{2}-{g^{\prime }}_{1}}{\eta g_{2}-g_{1}}dx=\infty$. Then *F* is uniquely determined by the functions $g_{1},\;g_{2}$ and η. Furthermore, the density function of *F* is $f(x)\propto \frac{\eta^{\prime }}{\eta g_{2}-g_{1}}\exp\left\{-\int_{a}^{x}\frac{\eta^{\prime }g_{2}}{\eta g_{2}-g_{1}}dt\right\}$.

The following theorem provides a characterization for the *EE*-*X* family of distributions in (1.5).

Theorem 5 Let Y:Ω→H be a continuous random variable. Then *Y* follows the *EE*-*X* family in (1.5) if and only if the functions in Theorem 4 can be chosen as: $\eta =0.5{\left(1-F\right)}^{\lambda }$, $g_{1}=g_{2}{\left(1-F\right)}^{\lambda }$ and $g_{2}={\left(1-{\left(1-F\right)}^{\lambda }\right)}^{1-\alpha }$, x∈H;α,λ>0, where *F* is the CDF of the random variable *X* defined in (1.5).

Proof Using (1.5), one can show $e_{g_{1}}(x)=0.5\alpha {\left(1-F(x)\right)}^{2\lambda -1}$ and $e_{g_{2}}(x)=\alpha {\left(1-F(x)\right)}^{\lambda -1}$. Therefore, $\eta (x)g_{2}(x)-g_{1}(x)=-0.5{\left(1-F(x)\right)}^{\lambda }g_{2}(x)<0$. This implies that the equation $\eta g_{2}-g_{1}=0$ has no solution on the interior of *H*. Also, it is not hard to show that $\int_{a}^{b}\frac{\eta {g^{\prime }}_{2}-{g^{\prime }}_{1}}{\eta g_{2}-g_{1}}dx=\infty$. Since other assumptions of Theorem 4 are obvious, *Y* has the density function in (1.5). Furthermore, $\frac{\eta^{\prime }g_{2}}{\eta g_{2}-g_{1}}=\lambda h_{f}(x)$ which implies that $g(y)\propto \frac{h_{f}(x)}{g_{2}(x)}\exp\{-\lambda H_{f}(x)\}$ where the normalizing constant can be determine easily as λα. □

Corollary 2 Let Y:Ω→H be a continuous random variable. Let $g_{2}={\left(1-{\left(1-F\right)}^{\lambda }\right)}^{1-\alpha }$, x∈H;α,λ>0. Then *Y* follows the *EE*-*X* family in (1.5) if and only if there exist functions $\eta =0.5{\left(1-F\right)}^{\lambda }$ and $g_{1}=g_{2}{\left(1-F\right)}^{\lambda }$ satisfying the differential $\frac{\eta^{\prime }(x)g_{2}(x)}{\eta (x)g_{2}(x)-g_{1}(x)}=\lambda h_{f}(x),\;\;x\in H.$

Corollary 3 Let Y:Ω→ℝ be a continuous random variable. Then *Y* follows the EECD if and only if the functions in Theorem 4 can be chosen as: $\eta =0.5{\left(1-F\right)}^{\lambda }$, $g_{1}=g_{2}{\left(1-F\right)}^{\lambda }$ and $g_{2}={\left(1-{\left(1-F\right)}^{\lambda }\right)}^{1-\alpha }$, x∈ℝ, where $F(x)=\frac{1}{2}+\frac{1}{\pi }\tan^{-1}\left(x/\theta \right)$ for α, λ, θ>0.

Theorem 6 Let X:Ω→H be a continuous random variable with CDF F(x). Let ψ(x) be a differentiable function defined on *H* such that $\lim_{x\to \infty }\psi (x)=1$. Then for δ≠1, E[ψ(x)|X<x]=δψ(x) if and only if $\psi (x)=F^{\frac{1}{\delta }-1}(x),\;\;\;x\in H$.

Proof See Domma and Hamedani [27]. □

Corollary 4 Let Y:Ω→H be a continuous random variable. If $\psi (x)=1-{\left(1-F(x)\right)}^{\lambda },\;\;\;x\in H$ and δ=α/(α+1) then Theorem 6 gives the CDF of the *EE*-*X* family in (1.5).

## Parameter estimation

### Maximum likelihood estimation method (MLE)

Let a random sample of size *n* be taken from the EEC distribution. The log-likelihood function for the EEC distribution in (2.1) is given by

(4.1) $$\log L(\alpha ,\lambda ,\theta )=\sum_{i=1}^{n}\log g(x_{i};\alpha ,\lambda ,\theta )=n\log\alpha +n\log\lambda -n\log\pi +n\log\theta -\sum_{i=1}^{n}\log(\theta^{2}+x_{i}^{2})+(\alpha -1)\sum_{i=1}^{n}\log(1-z_{i}^{\lambda })+(\lambda -1)\sum_{i=1}^{n}\log(z_{i}),$$

where $z_{i}=0.5-\pi^{-1}\tan^{-1}(x_{i}/\theta )$.

The MLE $\hat{\alpha }$, $\hat{\lambda }$ and $\hat{\theta }$ can be obtained by maximizing the log likelihood function in (4.1) numerically. The initial value for θ is taken to be the MLE of θ by assuming the data, $x_{1},x_{2},\cdots ,x_{n}$, follows the Cauchy distribution. The initial values for the parameters α and λ are taken as follows: From Remark 1, the initial values of α and λ are taken to be the MLEs of α and λ by assuming the data $y_{i}=\theta_{0}\cot(\pi e^{-x_{i}}),\;\;i=1,\cdots ,n$ follows EE(α,β). PROC NLMIXED in SAS is used to maximize the log-likelihood function in (4.1). In addition to the goodness of fit statistics, PROC NLMIXED gives the parameter estimates with their standard errors, which are the square roots of the diagonal entries in the estimated covariance matrix.

### Alternative method of moment estimation (AMM)

Since the moments of the EECD do not always exist, we consider in this section an alternative method of moment estimation which was first proposed by Zografos and Balakrishnan [28].

Theorem 7 If *X* follows EECD with parameters α, λ and θ, then for any r∈N, $E{\left(1-F(X)\right)}^{r}=\alpha B(\alpha ,1+r/\lambda )$, where $F(x)=2^{-1}+\pi^{-1}\tan^{-1}(x/\theta )$ is the CDF of the Cauchy distribution with parameter θ. □

Proof Straightforward and hence omitted.

Using Theorem 7, and by equating the corresponding sample moments with the population moments we have the following three equations

(4.2) $$\frac{1}{n}\sum_{i=1}^{n}{\left[2^{-1}+\pi^{-1}\tan^{-1}(x_{i}/\theta )\right]}^{r}=\alpha B(\alpha ,1+r/\lambda ),\;\;\;\;\;r=1,2,3.$$

The alternative method of moments estimates $\tilde{\alpha },\;$$\tilde{\lambda }$ and $\tilde{\theta }$ are obtained by solving the equations in (4.2) iteratively.

### Simulation study

To evaluate the performance of the MLE and the AMM methods, a simulation study for both methods is conducted for a total of five parameter combinations and the process is repeated 1000 times. Three different sample sizes *n* = 50, 70 and 100 are considered. The bias (estimate-actual), and the mean square errors (MSE) of the parameter estimates for the MLE and the AMM are presented in Table 1, Table 2 respectively. From the results in Table 2, it appears that the mean square errors for some parameters using the AMM method are unacceptably high. This can be seen more clearly for the parameter θ. The results in Table 1 show that the ML estimates, in most cases, have smaller mean square errors than the AMM estimates. Also, the bias using MLE method is acceptable. These results suggest using the MLE methods for data fitting. Also, a close look at the results from the small simulation study in Table 1, it is noticed that when α≤1(α>1), the MLE of α is overestimated (underestimated). Also, when α≥1(α<1), the MLE of λ is overestimated (underestimated). Furthermore, Table 1 indicates that the MLE of θ is always overestimated.

**Table 1:** Bias and standard deviation of the parameter estimates using MLE method.

Sample size	Actual values			Bias			MSE		
*n*	α	λ	θ	$\hat{\alpha }$	$\hat{\lambda }$	$\hat{\theta }$	$\hat{\alpha }$	$\hat{\lambda }$	$\hat{\theta }$
50	1	1	1	0.7997	0.2018	0.1785	0.7629	0.2460	0.2744
	1.5	0.5	1	−0.4319	0.4147	0.1799	0.6385	0.2669	0.2126
	1.5	1	2	−0.4231	0.7412	0.2556	0.6271	0.7634	0.5960
	0.8	0.5	0.7	0.3368	−0.0354	0.0945	0.1777	0.0138	0.0769
	0.6	1.2	2	0.4837	−0.4128	0.1791	0.2974	0.2052	0.4396

70	1	1	1	0.1334	0.1070	0.1010	0.1231	0.0874	0.1033
	1.5	0.5	1	−0.3842	0.3256	0.0908	0.2269	0.1462	0.1002
	1.5	1	2	−0.3760	0.6214	0.1448	0.2586	0.5113	0.3439
	0.8	0.5	0.7	0.2848	−0.0607	0.0557	0.1199	0.0111	0.0491
	0.6	1.2	2	0.4855	−0.4117	0.1766	0.2858	0.1981	0.3403

100	1	1	1	0.1111	0.0946	0.0691	0.0641	0.0523	0.0590
	1.5	0.5	1	−0.2347	0.3052	0.0514	0.2340	0.1205	0.0591
	1.5	1	2	−0.2369	0.5838	0.0825	0.2496	0.4299	0.2307
	0.8	0.5	0.7	0.2449	−0.0814	0.0261	0.0837	0.0112	0.0264
	0.6	1.2	2	0.4594	−0.4430	0.0866	0.2513	0.2184	0.2245

**Table 2:** Bias and standard deviation of the parameter estimates using AMM.

Sample size	Actual values			Bias			MSE		
*n*	α	λ	θ	$\hat{\alpha }$	$\hat{\lambda }$	$\hat{\theta }$	$\hat{\alpha }$	$\hat{\lambda }$	$\hat{\theta }$
50	1	1	1	0.1709	0.0484	0.2244	0.6445	0.3925	0.9889
	1.5	0.5	1	−0.4835	0.6667	0.3531	0.7893	0.7783	1.3293
	1.5	1	2	0.1853	−0.0305	0.0519	1.6612	0.4617	2.8034
	0.8	0.5	0.7	−0.3502	0.5290	0.0932	0.1716	0.5116	0.2202
	0.6	1.2	2	0.0707	−0.3663	−0.2680	0.1972	0.4504	1.7952

70	1	1	1	0.1324	0.0026	0.1113	0.5628	0.2894	0.5619
	1.5	0.5	1	−0.6535	0.5351	0.1123	0.6377	0.5021	0.4480
	1.5	1	2	0.0709	−0.0544	0.0900	1.0081	0.3206	2.3761
	0.8	0.5	0.7	−0.3379	0.5733	0.1366	0.1534	0.5595	0.2098
	0.6	1.2	2	0.0457	−0.4095	−0.3439	0.2303	0.5065	1.3382

100	1	1	1	0.0625	−0.0463	0.0462	0.4782	0.2568	0.4878
	1.5	0.5	1	−0.6484	0.5646	0.1527	0.5515	0.5134	0.4186
	1.5	1	2	0.0022	−0.0853	−0.0700	0.7371	0.2513	1.2762
	0.8	0.5	0.7	−0.3850	0.4892	0.0609	0.1807	0.4212	0.1719
	0.6	1.2	2	−0.0120	−0.4529	−0.4426	0.1403	0.4586	1.3005

## Application

To illustrate the applications of the EEC distribution, the EEC distribution is fitted to two data sets. The first data set in Table 3 (http://www.ibge.gov.br/seriesestatisticas/exibedados.php?idnivel=BR&idserie=PRECO101), is the INPC data which represents the national index of consumer prices of Brazil since 1979. The INPC index measures the cost of living of households with heads employees. The second data set in Table 4 from Weisberg [29], represents the sum of skin folds in102 male and 100 female athletes collected at the Australian Institute of Sports. The data sets are fitted to the EEC distribution and compared with the two-parameter Cauchy, the three parameter skew-Cauchy [8] and the beta-Cauchy [10] distributions. The following are the PDF of the Cauchy, skew-Cauchy and beta-Cauchy distributions respectively;

$$g(x)=\frac{1}{\pi \theta [1+{\left((x-c)/\theta \right)}^{2}]},\;x\in \mathbb{R}.$$

$$g(x)=\frac{1}{\pi \theta [1+{\left((x-c)/\theta \right)}^{2}]}\left(1+\frac{\lambda (x-c)}{\sqrt{\theta^{2}+(1+\lambda^{2}){\left(x-c\right)}^{2}}}\right),\;x\in \mathbb{R}.$$

$$g(x)=\frac{1}{\pi \theta B(\alpha ,\lambda )[1+{\left((x-c)/\theta \right)}^{2}]}{\left(\frac{1}{2}+\frac{1}{\pi }\tan^{-1}(x/\theta )\right)}^{\alpha -1}{\left(\frac{1}{2}-\frac{1}{\pi }\tan^{-1}((x-c)/\theta )\right)}^{\lambda -1},\;x\in \mathbb{R}.$$

**Table 3:** The INPC data.

0.69	0.44	0.13	0.03	0.17	0.37	2.47	0.62	0.57	1.39	0.39
0.97	0.42	0.12	−0.11	0.50	0.39	2.70	0.31	0.84	0.30	0.55
0.43	0.49	0.27	0.70	0.73	0.82	3.39	1.07	0.48	−0.05	0.74
0.30	0.62	0.23	0.91	0.50	0.18	1.57	0.74	0.49	0.09	0.07
0.25	0.42	0.38	0.73	0.40	0.04	0.83	1.29	0.77	0.13	0.05
0.59	0.43	0.40	0.44	0.41	−0.06	0.86	0.94	0.55	0.05	0.47
0.32	0.16	0.54	0.57	0.57	0.99	1.15	0.44	0.29	0.61	1.28
0.31	−0.02	0.58	0.86	0.39	1.38	0.61	0.79	0.16	0.74	1.29
0.26	0.11	0.15	0.44	0.83	1.37	0.09	1.11	0.43	0.94	0.65
0.26	−0.07	0.00	0.17	0.54	1.46	0.68	0.60	1.21	0.96	0.42
−0.18	−0.28	0.49	0.15	0.18	0.68	0.34	1.20	0.29	1.51	2.46
0.11	0.15	0.54	0.29	0.35	0.45	0.38	1.33	0.71	1.40	2.18
−0.31	0.72	0.85	0.10	0.11	0.81	0.02	1.28	1.46	1.17	2.10
−0.49	0.45	0.57	−0.03	0.60	0.33	0.50	0.93	1.65	1.02	2.49
1.62	1.01	1.44

**Table 4:** The sum of skin folds data.

28.0	98	89.0	68.9	69.9	109.0	52.3	52.8	46.7	82.7	42.3
109.1	96.8	98.3	103.6	110.2	98.1	57.0	43.1	71.1	29.7	96.3
102.8	80.3	122.1	71.3	200.8	80.6	65.3	78.0	65.9	38.9	56.5
104.6	74.9	90.4	54.6	131.9	68.3	52.0	40.8	34.3	44.8	105.7
126.4	83.0	106.9	88.2	33.8	47.6	42.7	41.5	34.6	30.9	100.7
80.3	91.0	156.6	95.4	43.5	61.9	35.2	50.9	31.8	44.0	56.8
75.2	76.2	101.1	47.5	46.2	38.2	49.2	49.6	34.5	37.5	75.9
87.2	52.6	126.4	55.6	73.9	43.5	61.8	88.9	31.0	37.6	52.8
97.9	111.1	114.0	62.9	36.8	56.8	46.5	48.3	32.6	31.7	47.8
75.1	110.7	70.0	52.5	67	41.6	34.8	61.8	31.5	36.6	76.0
65.1	74.7	77.0	62.6	41.1	58.9	60.2	43.0	32.6	48	61.2
171.1	113.5	148.9	49.9	59.4	44.5	48.1	61.1	31.0	41.9	75.6
76.8	99.8	80.1	57.9	48.4	41.8	44.5	43.8	33.7	30.9	43.3
117.8	80.3	156.6	109.6	50.0	33.7	54.0	54.2	30.3	52.8	49.5
90.2	109.5	115.9	98.5	54.6	50.9	44.7	41.8	38.0	43.2	70.0
97.2	123.6	181.7	136.3	42.3	40.5	64.9	34.1	55.7	113.5	75.7
99.9	91.2	71.6	103.6	46.1	51.2	43.8	30.5	37.5	96.9	57.7
125.9	49.0	143.5	102.8	46.3	54.4	58.3	34.0	112.5	49.3	67.2
56.5	47.6	60.4	34.9							

The maximum likelihood estimates, the log-likelihood value, the AIC (Akaike Information Criterion), the Kolmogorov-Smirnov (K-S) test statistic, and the *p*-value for the K-S statistic for the fitted distributions to the data sets are reported in Table 5, Table 6.

The results in Table 5, Table 6 show that the Cauchy distribution does not provide adequate fit to both data sets. The Skew-Cauchy distribution does not provide adequate fit to data sets in Table 4 and provides adequate fit to the data in Table 3. The EEC and beta-Cauchy distributions provide the best fit to the two data sets. The fact that EECD has less number of parameters compared with beta-Cauchy distribution makes EECD a better choice for fitting both data sets.

**Table 5:** Parameter estimates for the INPC data.

Distribution	Beta-Cauchy	EEC	Skew Cauchy	Cauchy
Parameter Estimates	$\hat{c}$ = −0.0226 a(0.2279)	$\hat{\theta }$ = 0.9949 (0.2891)	$\hat{c}$ = 0.2424 (0.0818)	$\hat{c}$ = 0.4792 (0.0323)
	$\hat{\theta }$ = 0.7064 (0.1595)	$\hat{\alpha }$ = 27.3016 (18.5834)	$\hat{\theta }$ = 0.3275 (0.0531)	$\hat{\theta }$ = 0.2656 (0.0285)
	$\hat{\alpha }$ = 9.3393 (5.2784)	$\hat{\lambda }$ = 3.4706 (0.9507)	$\hat{\lambda }$ = 1.1888 (0.5256)	
	$\hat{\lambda }$ = 4.0236 (1.6028)			

$\hat{\ell }$	−116.5629	−116.7820	−132.7465	−139.3542
AIC	241.1257	239.5641	271.4929	282.7083
K-S	0.0376	0.0372	0.0837	0.1115
K-S *p*-value	0.9793	0.9815	0.2219	0.0403

a Standard error.

**Table 6:** Parameter estimates for the sum of skin folds data.

Distribution	Beta-Cauchy	EEC	Skew Cauchy	Cauchy
Parameter Estimates	$\hat{c}$ = 11.7939 (6.1381)	$\hat{\theta }$ = 15.9717 (16.6657)	$\hat{c}$ = 30.1404 (0.4983)	$\hat{c}$ = 55.5789 (1.9777)
	$\hat{\theta }$ = 19.4238 (6.1543)	$\hat{\alpha }$ = 666.6127 (128.0986)	$\hat{\theta }$ = 27.9345 (2.6184)	$\hat{\theta }$ = 16.9283 (1.6372)
	$\hat{\alpha }$ = 26.5961 (9.1507)	$\hat{\lambda }$ = 2.7858 (0.3756)	$\hat{\lambda }$ = 29.6768 (18.5649)	
	$\hat{\lambda }$ = 4.0223 (1.0913)			

$\hat{\ell }$	−955.0111	−955.7381	−972.6959	−1011.7310
AIC	1918.0220	1917.4760	1951.3920	2027.4630
K-S	0.0760	0.0700	0.1352	0.1794
K-S *p*-value	0.1937	0.2758	0.0012	0.0000

From Table 5, the estimated value for the parameter θ for the EEC distribution is approximately 1. Therefore, the two-parameter EEC distribution can be a natural choice for this data set. The likelihood ratio test for the hypothesis $H_{0}:\theta =1$ against $H_{a}:\theta \neq 1$ confirms that the two-parameter EEC (standard EEC) distribution performs equally well when compared with the three-parameter EEC distribution. The results from fitting the standard EEC distribution to the INPC data as follows:

$$\begin{array}{c} \hat{\alpha }=27\;.6086(5.9527),\;\;\;\hat{\lambda }=3.4865(0.2311),\;\;\;\overset{}{\hat{\ell }}=-116.7822,\\ AIC=237.5644,\;\;\;\;\text{K-S}=0.0375\;\;\;\;and\;\;\;\;\mathrm{K-S p}\text{-value}=0.9799. \end{array}$$

**Fig. 7:**
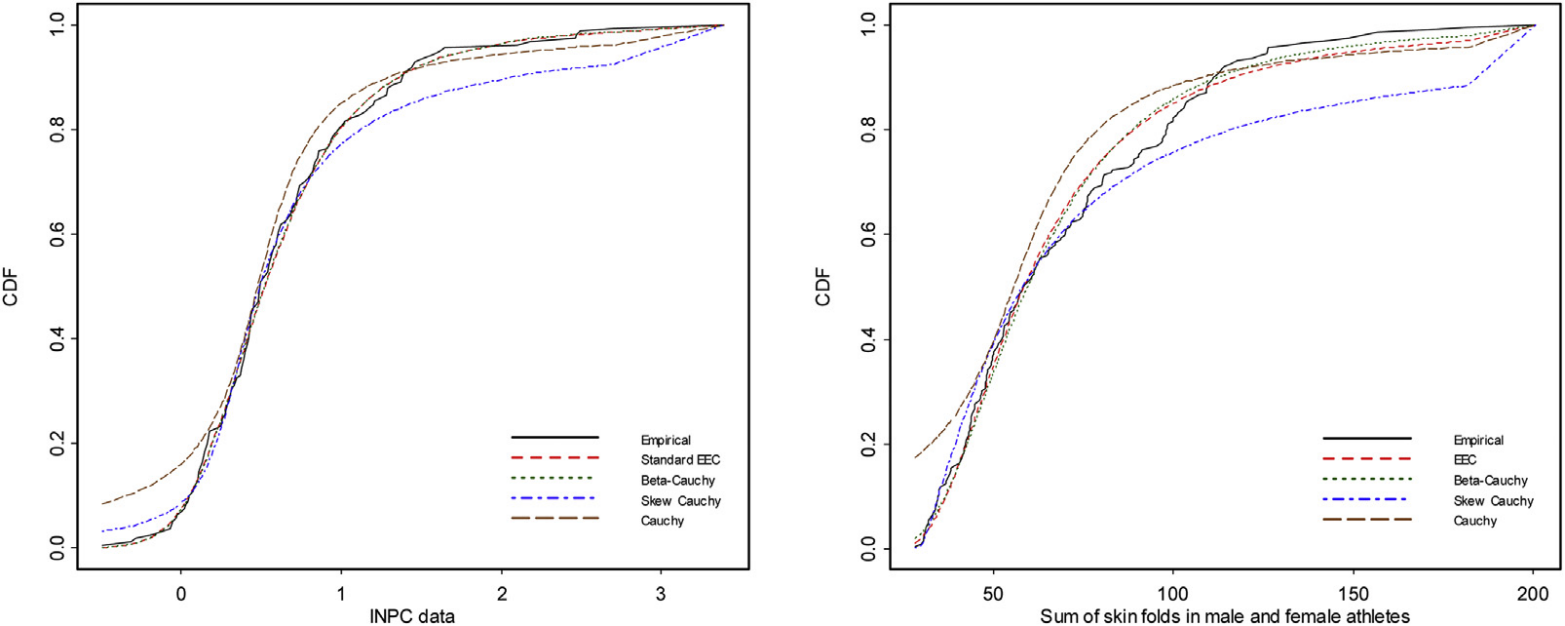
CDF for fitted distributions for data sets in Table 5, Table 6.

The likelihood ratio statistic in this case is based on $\lambda =L_{0}(\tilde{\alpha },\tilde{\lambda })/L_{a}(\hat{\alpha },\tilde{\lambda },\tilde{\theta })$, where $L_{0}$ and $L_{a}$ are the likelihood values for the standard EEC and the three-parameter EEC distributions respectively. The quantity −2logλ asymptotically follows a chi-square distribution with 1 degree of freedom. In this case, we have −2logλ=0.0004 and the *p*-value is 1.0000. Fig. 7 displays the empirical and the fitted cumulative distribution functions for the data sets in Table 3, Table 4. In this Figure, the standard EEC distribution is used for the data set in Table 3 and the three-parameter EEC distribution is used for the data set in Table 4. The figure supports the results from Table 5, Table 6.

## Summary and conclusion

In this article, a generalization of the Cauchy distribution, the EECD, is defined and studied. Several properties of the proposed distribution are studied in detail including mode, moments, skewness, kurtosis and Shannon entropy. Two real data sets are fitted to the EECD and compared with Cauchy, skew Cauchy and beta-Cauchy distributions. The results show the great flexibility of the proposed model. Based on Fig. 4, Fig. 5, the EEC indeed can fit different data sets with wide range of skewness values including left and right skewness. Furthermore, a simulation study is conducted for various parameter and sample size values to generate highly left and right skewed data sets from EECD. The results, based on K-S statistic, showed that the EECD produces good fit to various highly skewed (left and right) data sets. To conserve space, the results were not included in the article.

For future research, one can propose methods of discrimination between two or more members of the *EE*-*X* family based on the ratio of the Shannon entropies. For more information about this problem, one is referred to Zografos and Balakrishnan [28]. Furthermore, one can use the kullback-Leibler divergence [30] to discriminate between member of *EE*-*X* family and other family such as the beta family [11]. Also, it is noteworthy to mention that the method of discrimination between members of *EE*-*X* family using the idea proposed by Zografos and Balakrishnan [28] can be extended to cover the gamma-*X* family [13] or even the *T*-*X* family [12].
